# Intelligent viewpoint selection for efficient CT to video registration in laparoscopic liver surgery

**DOI:** 10.1007/s11548-017-1584-7

**Published:** 2017-04-11

**Authors:** Maria R. Robu, Philip Edwards, João Ramalhinho, Stephen Thompson, Brian Davidson, David Hawkes, Danail Stoyanov, Matthew J. Clarkson

**Affiliations:** 1 Centre For Medical Image Computing, Engineering Front Building, University College London, Malet Place, London, UK; 2 Royal Free Campus, UCL Medical School, 9th Floor, Royal Free Hospital, Rowland Hill Street, London, UK

**Keywords:** Gaussian curvature, Image guidance, Laparoscopic liver surgery, Rigid registration, View planning

## Abstract

**Purpose:**

Minimally invasive surgery offers advantages over open surgery due to a shorter recovery time, less pain and trauma for the patient. However, inherent challenges such as lack of tactile feedback and difficulty in controlling bleeding lower the percentage of suitable cases. Augmented reality can show a better visualisation of sub-surface structures and tumour locations by fusing pre-operative CT data with real-time laparoscopic video. Such augmented reality visualisation requires a fast and robust video to CT registration that minimises interruption to the surgical procedure.

**Methods:**

We propose to use view planning for efficient rigid registration. Given the trocar position, a set of camera positions are sampled and scored based on the corresponding liver surface properties. We implement a simulation framework to validate the proof of concept using a segmented CT model from a human patient. Furthermore, we apply the proposed method on clinical data acquired during a human liver resection.

**Results:**

The first experiment motivates the viewpoint scoring strategy and investigates reliable liver regions for accurate registrations in an intuitive visualisation. The second experiment shows wider basins of convergence for higher scoring viewpoints. The third experiment shows that a comparable registration performance can be achieved by at least two merged high scoring views and four low scoring views. Hence, the focus could change from the acquisition of a large liver surface to a small number of distinctive patches, thereby giving a more explicit protocol for surface reconstruction. We discuss the application of the proposed method on clinical data and show initial results.

**Conclusion:**

The proposed simulation framework shows promising results to motivate more research into a comprehensive view planning method for efficient registration in laparoscopic liver surgery.

## Introduction

Approximately 1800 liver resections are performed annually in the UK for primary or metastatic surgery. However, this is a major global health problem and more than 150,000 patients per year could benefit from liver resection. Augmented reality has been shown [12] to aid in surgical interventions through improved resection quality and a reduction in positive surgical margins. Such image guidance systems could make surgeons reconsider the suitable cases for MIS, thus increasing the number of patients.

The 3D–3D registration between a liver model derived from the CT scan and the laparoscopic data could be performed with a surface reconstruction of the surgical scene. Even though they achieve promising results, most methods [4, 8, 20, 27] are applied in open surgery and acquire the surgical environment with a laser range camera to get a point cloud. In the context of minimally invasive surgery, one approach is to capture with the laparoscopic camera as many surface patches as possible in order to get a reasonable reconstruction. In the SmartLiver system [28], a user manually selects surface patches to be non-overlapping and well distributed on the visible part of the liver. However, these selections are user dependent and there is no specific protocol to follow. Furthermore, this step is usually performed with the assistance of technical staff who have to be present at all the surgical interventions. Another approach consists of an initial exploratory video [5] in which the clinician moves the laparoscopic camera for several seconds around the organ of interest, while their algorithm selects the sharp images in the background. However, not all the collected patches contribute equally to the final data fusion. We propose the reformulation of view planning for the purpose of an efficient registration.

## State of the art

The concept of view planning was introduced in robotics where large scanning sensors had to be moved around a physical object until a complete 3D reconstruction was achieved. So, view planning algorithms propose solutions that determine a suitably short list of best views that would output an acceptable 3D model under the constraints of the imaging environment. A good survey of existing techniques in view planning can be found in [23]. A wide range of research fields have integrated the use of view planning related concepts to automate and optimise their workflow—scene exploration [6, 13], object recognition [1, 7, 17, 24], scene inspection [18, 25]. In this paper, we reformulate view planning in the scope of registration in minimally invasive surgery. Hence, an optimal subset of viewpoints has to be collected in order to maximise the accuracy of the registration between an a priori model and the surface reconstruction.

Closely related techniques have been published by [16, 26]. The doctoral work of David Simon [26] looks at achieving high accuracy registration with experiments conducted in open surgery. His proposed framework was based on the observation that intelligent data selection (IDS) from specific locations can lead to much better registration than acquiring large and random collections of data. So, he proposed analysing the constraint imposed by a surface on a rigid transformation. Ma et al. [15] extended Simon’s approach by providing an alternative derivation for his constraint analysis which tackles several of its limitations. Ma et al. [16] later incorporated their analysis in a unified method for registration and point selection using a particle filter.

There are several aspects in which the methods proposed by [16, 26] differ from what we aim to develop. Both methods use a digitising probe to acquire the data points during the surgery. Simon’s approach filters out high curvature points, since it would be difficult for a user to acquire them with precision. Thus, the algorithm only suggests data in flat regions which can be more accurately represented by an uncertainty area around them. While their filtering technique shows better results for their chosen registration metric, we plan to use exactly the high curvature points to constrain the registration. This approach stems from the intuition that highly distinctive geometrical areas could potentially achieve a more efficient registration. Secondly, we plan to explicitly include in our framework the constraints of the imaging environment—from the camera space to the clinically visible parts of the object. Furthermore, their proposed data collection methods are heavily influenced if the view of the intra-operative scene is restricted and only a small number of points are reachable. Consequently, their method would be impractical and difficult to integrate in a minimally invasive scenario.

Other related methods have been proposed by [9, 14, 21]. Low et al. [14] propose an ICP registration predictor which can output absolute error bounds and use it as a selection criterion for view planning in object reconstruction for range acquisition of indoor environments. Rusinkiewicz and Levoy [21] propose a point selection strategy for ICP which maximises the distribution of normals in a sphere. Gelfand et al. [9] extend the method to maximise both translational and rotation constraints on the transformation.

A different approach to investigate the registration error variation with respect to different data collection strategies was proposed by [30, 31] in CT based navigation on the vertebra and neuronavigation, respectively. They repeatedly select points in different configurations and simulate hundreds of registrations to observe the correlation between the regions with the registration accuracy. While they highlight a specific protocol for the surgeons, the recommended configurations are difficult to generalise to other organs.

Finally, previous methods have investigated different metrics (i.e. visibility, view overlap) for a wide range of applications (i.e. object reconstruction, classification). We aim to investigate the feasibility of a view planning approach in registration in minimally invasive surgery. As stated in [26], increasing the amount of data points collected will typically improve the registration accuracy. However, when the time is limited, the need for an automatic method to generate an optimal registration increases. A clear concise protocol would also reduce user dependent variability in registration performance.

## Contribution of this paper

We focus on improving the efficiency of 3D–3D rigid registration of shapes—between a 3D liver model extracted from the pre-operative CT scan and the point cloud of a liver patch seen from a specific viewpoint. Furthermore, we are currently investigating rigid registration and its validation in a clinical setting, given the difficulty of assessing the performance of deformable methods.

We have developed a simulation framework in order to determine which viewpoints would represent the best constraints for registration. We propose to automatically select these views based on a scoring strategy in order to reach an optimal registration accuracy. This approach aims to remove the need for a timely exploratory video or for the reconstruction of a high number of patches. Therefore, it would make the data acquisition process during surgery faster, more intuitive and less disruptive for the surgical workflow. We explicitly include the constraints specific to camera movement and limited field of view to simulate a realistic surgical scene. In order to validate the feasibility of the proposed approach in laparoscopic surgery, we address the following questions:

- **How can we evaluate the viewpoints?** (“Viewpoint evaluation” section) We use synthetic data to validate whether the proposed scoring strategy is correlated with higher registration accuracy. Furthermore, we investigate using an intuitive visualisation which regions of the liver could potentially lead to better registrations.
- **Can view planning improve registration?** (“View planning for registration” section) Simulated intra-operative data is used to demonstrate that the proposed pre-operative planning step can lead to a correct registration convergence for larger initial misalignments.
- **How many patches are needed for a low registration error?** (“Effect of the number of patches on registration accuracy” section) We validate the need for an initial pre-operative planning stage in which to automatically select the most distinctive liver patches.
- **How can view planning be applied in a real clinical scenario?** (“Application in laparoscopic liver resection” section) Clinical data from one human patient undergoing a laparoscopic liver resection is used to illustrate the use of the proposed approach in a surgical scenario. We discuss the additional challenges encountered with clinical data and possible future directions.

## Methods

Let $P_f$ denote the fixed point cloud of the liver surface extracted from a pre-operative CT scan. The moving point cloud $P_m$ represents the surface reconstructed from laparoscopic video. Most approaches to registration minimise the distance between $P_f$ and $P_m$ transformed by *T*:

1 $$\begin{aligned} \underset{T}{\mathrm{min}} \sum || P_f - T(P_m) ||^2 \end{aligned}$$

We focus on choosing the optimal set of measurements for $P_m$ in order to make the registration more efficient. Hence, our proposed method is independent of the registration algorithm.

The next sections detail the core components of the simulation framework. The search space of the laparoscopic camera is modelled to simulate the environment of laparoscopic surgery where the field of view is limited (“Search space representation” section). Given a camera position, the visible liver patch as well as the maximum visible area can be generated (“Visible surface simulation” section). Once a liver patch is available, the corresponding viewpoint is evaluated based on a scoring strategy (“Viewpoint scoring strategy” section).

### Search space representation

The fixed point cloud $P_f$ is placed at the scene origin, and it is isotropically scaled to fit in a unit sphere. The search space of a camera looking at the object of interest represents all the possible locations from which it can capture images. A common approach in view planning is to use a virtual sphere surrounding the object on which the cameras can be placed. This approach provides an intuitive way to parametrise the camera location and orientation by working with the spherical coordinates: radius *r*, longitude $\theta$ and colatitude $\phi$ instead of 3 translations and 3 rotations. The variation in the radius *r* corresponds to the distance from the camera to the surface of the object. This assumption also restricts the camera orientation by aligning it with the centre of the object. The variation in the spherical angles $\theta$, $\phi$ corresponds to a different camera location on the enclosing sphere given by the radius *r*.

However, we want to incorporate the reduced surgical invasiveness specific to laparoscopic surgery. Hence, the space of possible camera locations is heavily constrained. We consider the main camera position to be where the trocar is situated in the patient’s abdomen, pointing towards the centre of the liver: $(r_c,\theta _c,\phi _c)$. Depending on the surgical procedure, the clinicians can provide a range of available motion as $(r_c \pm \Delta _r,\theta _c \pm \Delta _{\theta },\phi _c \pm \Delta _{\phi })$.

### Visible surface simulation

Given a camera position, the liver model is projected into the viewpoint’s space. In order to simulate a realistic surface reconstruction, the points corresponding to the back surface of the 3D point cloud are not considered.

Multiple 3D patches can be generated for all the possible camera positions in the constrained viewing space in order to obtain the maximum visible surface $P_{\mathrm{max}}$ by merging them. Due to the large number of points obtained, the point cloud is downsampled before any other computation is done.

**Fig. 1:**
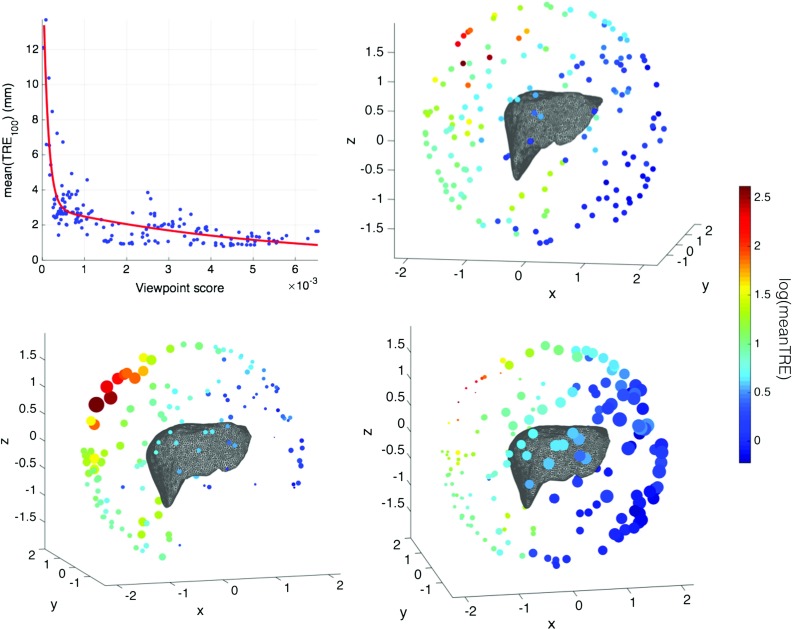
*Top left* correlation between the Gaussian curvature scoring strategy and the registration errors. *Top right* TRE variation over the liver surface. Each *sphere* represents a camera location. The colourmap is applied to *log*(*mean*(*TRE*)) over the 100 registrations for each sphere ($mean(TRE) \in [0.7992, 13.7048\,\mathrm{mm}]$). *Bottom left* the size of the spheres is proportional to *std*(*TRE*). So, a bigger variation in errors leads to larger spheres. *Bottom right* the size for the spheres is proportional to the curvature score $f_{\mathrm{surface}}$

### Viewpoint scoring strategy

We evaluate each liver patch in the constrained viewing space in order to rank them based on their surface quality. Consequently, the principal curvatures $k_1,k_2$ are approximated at each 3D point [2]. The score $f_{\mathrm{surface}}$ gives us an automatic way to distinguish between ambiguous and distinctive surfaces.

2 $$\begin{aligned} f_{\mathrm{surface}} = \frac{\sum \limits _i^{N} K_i }{N} \end{aligned}$$

where *N* is the number of points in each rendered scan and $$K_i = k_1^i k_2^i $$ is the approximated Gaussian curvature at each point.

**Fig. 2:**
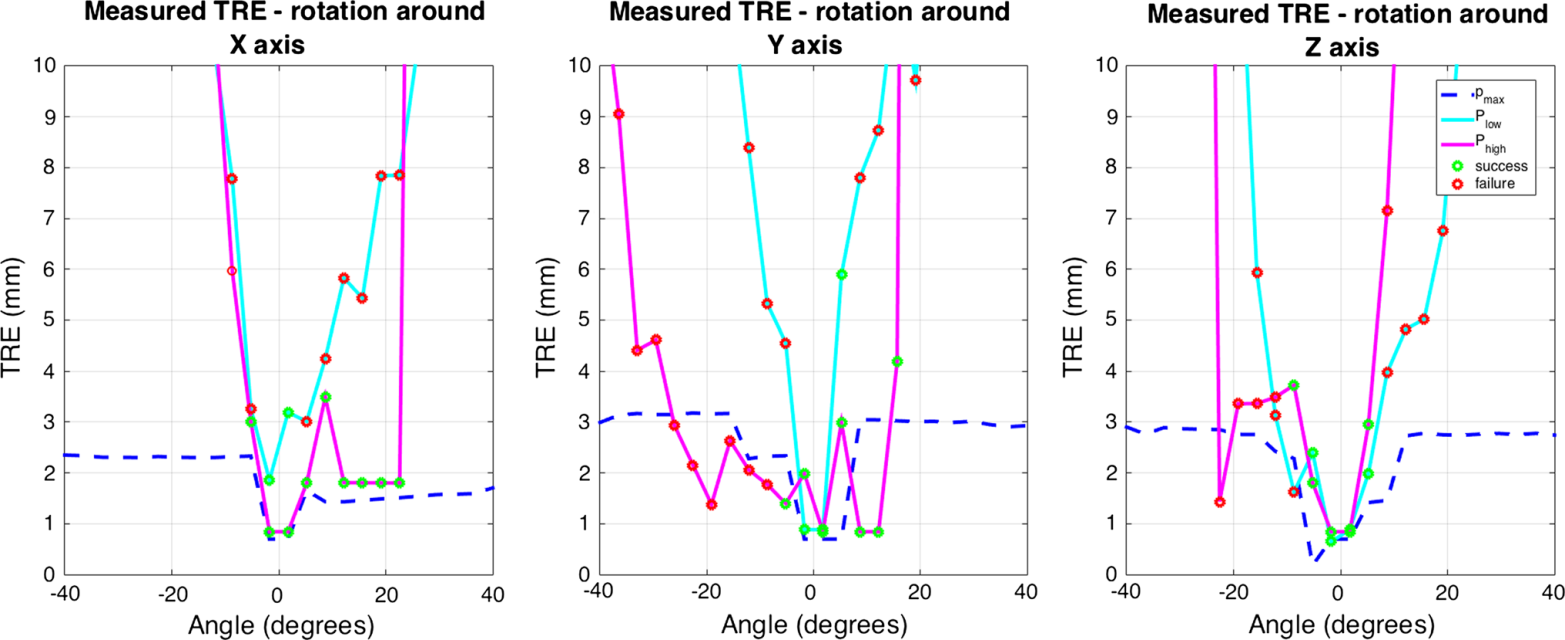
Basins of convergence for ICP for the lowest scoring point cloud $P_{\mathrm{low}}$, the highest scoring point cloud and $P_{\mathrm{high}}$ and the maximum visible surface $P_{\mathrm{max}}$. The *green circles* were omitted for clarity for the baseline as it always converges to the desired position

## Results

Experiments were performed to validate the feasibility of view planning for efficient registration in the context of laparoscopic liver surgery. The liver point cloud $P_f$ is extracted from a segmented CT scan of a human patient taken before the surgery.^1^ The point clouds $P_m$ used in the following experiments simulate what a camera would see intra-operatively, in a rigid scenario. Anisotropic noise [$~N(0,1\,\mathrm{mm})$] was added along the normals to $P_m$ in order to simulate the characteristic surface reconstruction noise. All the experiments use the iterative closest point (ICP) [3] algorithm for the registration step. A liver abnormality is simulated inside the model, and it is used as a landmark for measuring the target registration error (TRE). Note that these points are not used during the registration process with ICP.

**Fig. 3:**
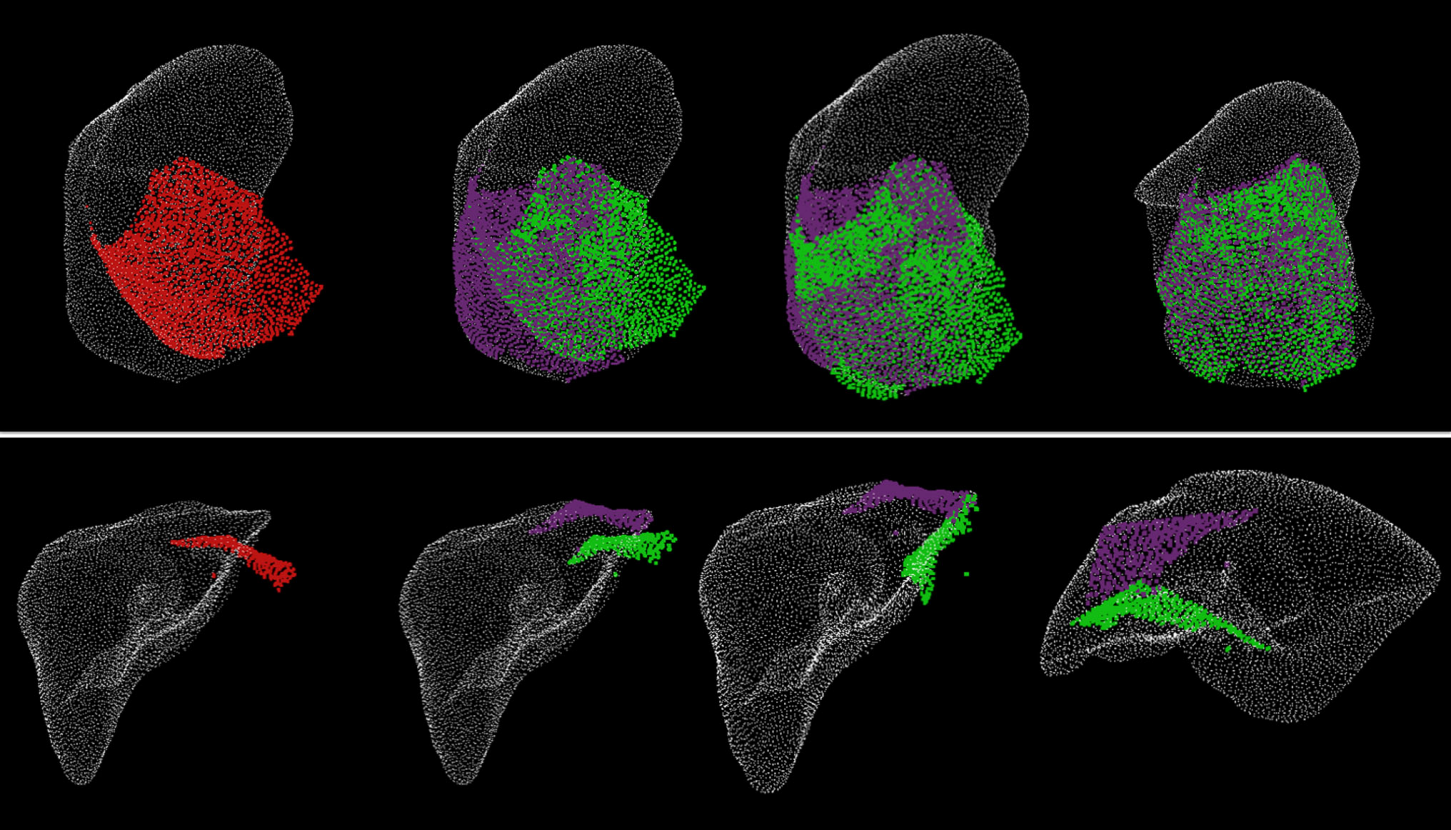
Visual assessment of ICP progress until convergence. *Colour coding*: fixed point cloud in *white*, moving point cloud after the rotation is applied in *red*, moving point cloud during ICP iterations in *green*, ideal final position of the moving point cloud in *purple*. *Top row* success example for a rotation of $40^\circ$ around the z axis for $P_\mathrm{max}$. The images from left to right show the iterations 0 (before ICP), 1, 12, 150. *Bottom row* failure example for a rotation of $18^\circ$ around the y axis for $P_{high}$. The images from left to right show the iterations 0 (before ICP), 1, 20, 25

All the experiments use a fixed radius for the enclosing sphere, thus further reducing to camera parametrisation from 6D to 2D. Consequently, the camera locations are specified using the spherical angles $\theta$, $\phi$ and the camera is always oriented towards the centre of the object. Furthermore, a range of $[-30^\circ ,30^\circ ]$ is used for the spherical coordinates in order to simulate the constrained search space (as described in “Viewpoint scoring strategy” section).

The simulation framework was implemented in C++ on a MacOS 10.11.2 laptop with an Intel Core i7 3.1 GHz processor. The project uses CMake (3.4.2) to enable compilation on multiple platforms. The libraries used as dependencies are: Eigen 3 [11] and the Point Cloud Library 1.2 (PCL) [22].

### Viewpoint evaluation

This experiment explores the distribution of registration errors for individual patches of the liver model. Since we are investigating which liver regions could lead to a good registration irrespective of the clinical case, we do not use any constraints on the viewing space for now. A total of 170 camera positions were randomly picked on a sphere enclosing the liver (with a constant radius). Given the camera location, a liver patch is rendered to simulate what the camera sees. Then, 100 transformations are generated by randomly choosing rotation and translation parameters ($rot_x, rot_y, rot_z, t_x, t_y, t_z$) in the ranges $rot \in [-10^{\circ }, 10^{\circ }]$ and $t \in [-10, 10\,\mathrm{mm}]$. These transformations are applied to each simulated liver patch and the target registration errors are recorded after ICP is used to recover their initial position. Figure 1 shows the distribution and variation of registration errors on the liver model.

### View planning for registration

This experiment analyses the basins of convergence of ICP between the original liver model $P_f$ and three different partial point clouds $P_m$ consisting of the patch with the lowest score $P_{\mathrm{low}}$, the patch with the highest score $P_{\mathrm{high}}$ and the maximum visible surface $P_{\mathrm{max}}$, respectively. The last point cloud is chosen as a best case scenario when the complete liver surface visible from a trocar position is reconstructed.

The basins of convergence are generated for rotations in the range $[-40^\circ ,40^\circ ]$ over 24 steps for each x, y, z axis. Since the visual assessment of the graphs (Fig. 2) makes it difficult to draw any clear conclusions about the differences between the scenarios, an interactive registration visualisation application was implemented. After the visual inspection of each case, the successful and failed cases have been overlaid on top of the graphs (Fig. 3).

It can be easily observed in Fig. 2 that the highest scoring view $P_{\mathrm{high}}$ recovers the correct transformation for larger misalignments (with more successful cases). The rotation around the x axis shows successful registrations for $P_{\mathrm{low}}$ between $[-2^\circ ,2^\circ ]$ , whereas for $P_{\mathrm{high}}$ the range goes to $[-6^\circ ,23^\circ ]$. The y rotation leads to a convergence in the global maximum for a range of $[-2^\circ ,6^\circ ]$ for $P_{\mathrm{low}}$ and $[-6^\circ ,16^\circ ]$ for $P_{\mathrm{high}}$. Similarly, for the z axis, the ranges are of $[-6^\circ ,6^\circ ]$ degrees for $P_{\mathrm{low}}$ and $[-9^\circ ,6^\circ ]$ degrees for $P_{\mathrm{high}}$. Interestingly, $P_{\mathrm{high}}$ has an asymmetrical convergence basin with respect to 0, which could be due to the irregular size of the patch. Furthermore, the plateau observed around 2.5 mm is a characteristic of the registration algorithm chosen (ICP). We propose the planning method as a separate component to be added to a registration pipeline, irrespective of the registration algorithm.

**Fig. 4:**
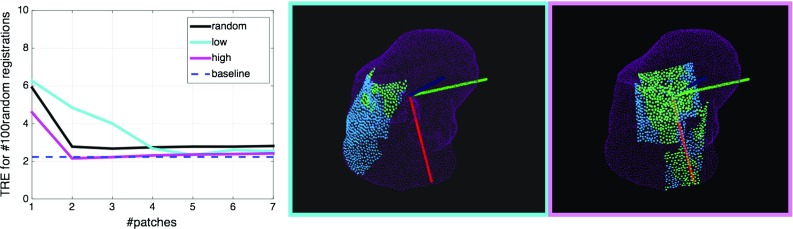
*Left* mean TRE variation (for 100 random transformations) with an increasing number of patches. *Middle* example of top 3 lowest scoring patches chosen by the algorithm. *Right* example of top 3 highest scoring patches. *Blue* regions highlight flat surfaces, whereas *green* symbolises positive Gaussian curvature

**Fig. 5:**
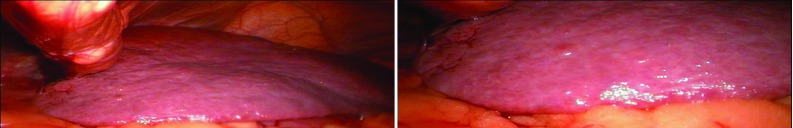
Example of video frames used for the stereo reconstruction of the surface in a laparoscopic liver resection

**Fig. 6:**
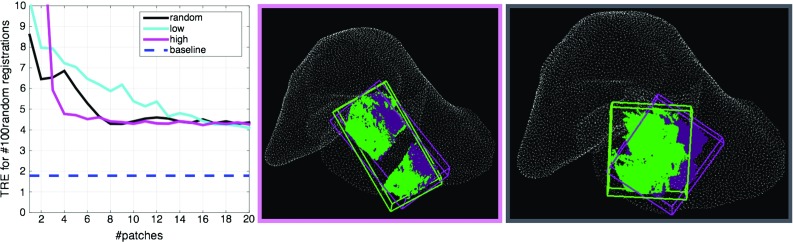
Visual assessment of registration with view planning for real data. *Colour coding*: fixed point cloud in *white*, moving point cloud in *green*, ideal final position given by a gold standard registration of the moving point cloud in *purple*. *Left* the previous experiment was re-ran for the constraints given by real data from a liver resection in order to suggest a number of patches to be considered. *Middle* registration results using clinical data after running ICP on a merged point cloud from the top 4 recommended patches by the proposed method. *Right* registration results using clinical data after running ICP on a merged point cloud from 4 random patches

**Fig. 7:**
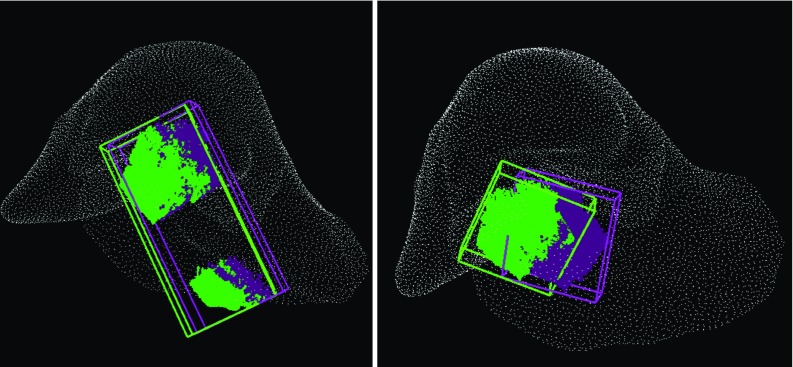
Registration examples highlighting the need for additional constraints in a real-world scenario. *Colour coding*: fixed point cloud in *white*, moving point cloud in *green*, ideal final position given by a gold standard registration of the moving point cloud in *purple*. *Left* the moving cloud is built from 2 patches—the highest scoring viewpoint and the patch which is furthest away from it. *Right* the moving point cloud is built from the top two highest scoring patches

### Effect of the number of patches on registration accuracy

This experiment explores the variation of the TRE with an increasing number of patches selected using each of the following strategies: random, top lowest scores and top highest scores. These are all compared to the maximum visible liver surface which is considered the baseline (Fig. 3, Top row).

Similarly to the previous experiment, 100 random transformations are generated and applied to the point cloud $P_m$ for each selection strategy. Once multiple patches are selected, they are merged into a point cloud which is subsequently used for registration.

Figure 4 shows that the two highest scoring views perform on average as well as the four lowest scoring views. Moreover, their errors are comparable to the ones obtained from the baseline with the registration of the maximum visible surface.

### Application in laparoscopic liver resection

In this experiment, we validate the proposed method on real data from one stereo video sequence from a liver resection (Fig. 5), which allows for a more realistic representation of intra-operative data. The liver is automatically segmented with the deep learning framework proposed in [10]. A total of 113 surface patches are reconstructed using [29] and merged together to build the maximal surface seen by the laparoscopic camera during surgery. So, we compare two scenarios for registration—the most distinctive patches suggested by the proposed method and a random subset.

Firstly, the proposed view planning pipeline is used to recommend the highest scoring camera positions based on the CT liver model of the patient. Given that ICP is used as the registration algorithm, a reasonably close initial alignment between the liver model and the camera space is needed. We employ a common approach [19, 28] in which this alignment is estimated by the user through the manual rotation and translation of the 3D model until it approximately matches the intra-operative surface reconstruction.

The constrained viewing sphere was built to match the maximal surface derived from the real surface reconstruction. We use a fixed radius of 30 mm, which represents the average distance of the laparoscope from the liver during the video. We use the previous experiment to decide on how many patches to select (see Fig. 6, left). The mean of the TRE for the 100 random perturbations applied to $P_{\mathrm{high}}$ goes down from 17.5 mm for one patch to 4.7 mm at 4 patches and seems to converge afterwards. Consequently, we use the first 4 suggested liver areas as the view planning output and we merge them to form $P_m$.

Secondly, we use our computation of the constrained viewing sphere to generate the random patches. This step is necessary to ensure that the two scenarios we are comparing have a similar covered surface area.

Once these simulated patches are recommended, we manually select the real surface patches that match them the most in position given an initial manual alignment between the CT and the reconstructed liver surface.

Figure 6 shows the visual assessment of the two considered scenarios—$P_m$ suggested by view planning and a randomly selected $P_m$. The random selection slides away from the correct position, whereas the views suggested by view planning appear to lock the registration close to the desired output.

## Discussion

Figure 1 motivates the use of the curvature score for view planning in registration. With higher scores in the more distinctive views, the registration error decreases rapidly. The scaling associated with the curvature score highlights the camera positions with low registration errors, at the same time reducing significantly the views with higher and more spread apart TRE values. Figure 1 allows an intuitive understanding of which liver regions lead to accurate registrations, as well as how to automatically choose them based on the curvature score. The graphs suggest the right lobe of the liver as being the most ambiguous for this camera space parametrisation. Similarly, the boundaries and the bottom side of the liver lead to the lowest errors.

Figure 2 points towards the feasibility of using view planning in the registration of intra-operative and pre-operative data. The explicit integration of high scoring patches in a registration algorithm could lead to a faster and more accurate registration for larger rotational offsets. Furthermore, these results are promising because they also show that for small enough rotations, similar registration errors could be achieved by both a high scoring patch and the maximum visible surface. This finding would remove the need for picking multiple patches to cover the whole area and change the focus to selecting the most distinctive patch. Such an approach could potentially automate the selection of useful patches, leading to less user interaction.

Intuitively, a partial point cloud that covers a larger portion of the surface should provide a better registration than a smaller region. However, we show in the third experiment (Fig. 4) that if the small view is distinctive enough, the target registration errors are comparable. Hence, the time spent collecting data could be significantly decreased by defining a laparoscopic camera guidance system.

Lastly, we compared our proposed view planning method with a random selection of patches on clinical data from a surface reconstruction in liver resection (Fig. 6). This experiment highlights the multiple challenges encountered in a real-world scenario: the deformation of the liver due to pneumoperitoneum, the flatness of the intra-operative surface and the noisy surface reconstruction. Due to these difficulties, we believe the proposed views in the last experiment perform better also because of the surface covered. Figure 7 highlights two scenarios with just two patches selected based on curvature and surface covered or based only on curvature. After 200 iterations, the selection based only on curvature starts to slide from the desired position, whereas the first selection performs better. We plan to further investigate how to incorporate additional constraints to arrive at an optimal selection suited to a laparoscopic scenario.

However, the proposed view planning approach has several limitations. The current implementation makes strong assumptions about the surgical scene (i.e. completely rigid, no motion from breathing or heartbeat) and the camera model (i.e. fixed field of view, fixed focal length, always looks at the centre of the liver). Nevertheless, a simplified yet relevant framework was needed to explore the feasibility of this approach in the context of abdominal image guidance. Given these promising results, more research will be done in this direction for the development of a comprehensive view planning technique for optimal registration.

Finally, one of the strengths of the proposed view planning simulation framework is that it can be easily made specific for surgical procedures. The clinician would have to input the trocar position and the maximum viewing space of the laparoscope, and the method would automatically suggest good views for efficient registration. An example of such an approach has been illustrated on real data from a liver resection. Such an intuitive protocol can be easily achieved by clinicians, avoiding the presence of any additional technical personnel during the surgical procedure. Furthermore, the reduction in the time spent acquiring data could potentially lead to surface reconstruction patches which are not as much influenced by heartbeat or breathing motion deformation.

## Conclusion

In this paper, we propose the use of view planning for a more efficient registration between a liver model derived from a pre-operative CT scan and laparoscopic data. We have shown its feasibility in a simulation environment of laparoscopic liver surgery and demonstrated it can be applied on clinical data. We conclude that intelligent viewpoint selection can provide an essential pre-operative planning tool for laparoscopic liver surgery. With further work, reduced operator dependence and registration time should be possible.

## Footnotes

1
www.visiblepatient.com.

## References

[1] Arbel T, Ferrie FP (2001). Entropy-based gaze planning. Image Vis Comput.

[2] Berkmann J, Caelli T (1994). Computation of surface geometry and segmentation using covariance techniques. IEEE Trans Pattern Anal Mach Intell.

[3] Besl PJ, McKay ND (1992). A method for registration of 3D shapes. IEEE Trans Pattern Anal Mach Intell.

[4] Clements LW, Chapman WC, Dawant BM, Galloway RL, Miga MI (2008). Robust surface registration using salient anatomical features for image-guided liver surgery: algorithm and validation. Med Phys.

[5] Collins T, Pizarro D, Bartoli A, Canis M, Bourdel N (2013) Realtime wide-baseline registration of the uterus in laparoscopic videos using multiple texture maps. In: Augmented reality environments for medical imaging and computer-assisted interventions, pp 162–171. doi:10.1007/978-3-642-40843-4_18

[6] Costante G, Forster C, Delmerico J, Valigi P, Scaramuzza D (2016) Perception-aware path planning. IEEE Trans Rob Autom, pp 1–16. http://arxiv.org/abs/1605.04151

[7] Deinzer F, Denzler J, Niemann H (2003). Viewpoint selection–planning optimal sequences of views for object recognition. CAIP.

[8] dos Santos TR, Seitel A, Kilgus T, Suwelack S, Wekerle AL, Kenngott H, Speidel S, Schlemmer HP, Meinzer HP, Heimann T, Maier-Hein L (2014). Pose-independent surface matching for intra-operative soft-tissue marker-less registration. Med Image Anal.

[9] Gelfand N, Ikemoto L, Rusinkiewicz S, Levoy M (2003) Geometrically stable sampling for the icp algorithm. In: 3DIM. IEEE, pp 260–267. doi:10.1109/IM.2003.1240258

[10] Gibson ED, Robu MR, Thompson SA, Edwards PE, Schneider C, Gurusamy K, Davidson BR, Hawkes DJ, Barrat DC, Clarkson MJ (2017) Deep residual networks for automatic segmentation of laparoscopic videos of the liver. In: SPIE, vol 10135, pp 101, 351M–101, 351M–6. doi:10.1117/12.2255975

[11] Guennebaud G, Jacob B (2010) Eigen v3. http://eigen.tuxfamily.org

[12] Hughes-Hallett A, Pratt P, Dilley J, Vale J, Darzi A, Mayer E (2015). Augmented reality: 3D image-guided surgery. Cancer Imaging.

[13] Kriegel S, Rink C, Bodenmuller T, Narr A, Suppa M, Hirzinger G (2012) Next-best-scan planning for autonomous 3D modeling. In: IROS, pp 2850–2856. doi:10.1109/IROS.2012.6385624

[14] Low KL, Lastra A (2007) Predetermination of icp registration errors and its application to view planning. In: 3DIM, pp 73–80. doi:10.1109/3DIM.2007.41

[15] Ma B, Ellis RE (2004) Spatial-stiffness analysis of surface-based registration. In: MICCAI, pp 623–630. doi:10.1007/978-3-540-30135-6_76

[16] Ma B, Ellis RE (2005) Unified point selection and surface-based registration using a particle filter. In: MICCAI, pp 75–82. doi:10.1007/11566465_10 10.1007/11566465_10 16685831

[17] Madsen C, Christensen H (1997). A viewpoint planning strategy for determining true angles on polyhedral objects by camera alignment. IEEE Trans Pattern Anal Mach Intell.

[18] Mason S (1997). Heuristic reasoning strategy for automated sensor placement. Photogramm Eng Remote Sens.

[19] Puerto-Souza GA, Cadeddu JA, Mariottini GL (2014). Toward long-term and accurate augmented-reality for monocular endoscopic videos. IEEE Trans Biomed Eng.

[20] Rucker DC, Wu Y, Clements LW, Ondrake JE, Pheiffer TS, Simpson AL, Jarnagin WR, Miga MI (2014). A mechanics-based nonrigid registration method for liver surgery using sparse intraoperative data. IEEE Trans Med Imaging.

[21] Rusinkiewicz S, Levoy M (2001) Efficient variants of the icp algorithm. In: 3DIM, pp 145–152. doi:10.1109/IM.2001.924423

[22] Rusu RB, Cousins S (2011) 3D is here: point cloud library (PCL). In: IEEE int conf robot autom. Shanghai, China. doi:10.1109/ICRA.2011.5980567

[23] Scott WR, Roth G, Rivest JF (2003). View planning for automated three-dimensional object reconstruction and inspection. CSUR.

[24] Shang L, Greenspan M (2010) Real-time object recognition in sparse range images using error surface embedding. Int J Comput Vis 89(2–3):211–228. doi:10.1007/s11263-009-0276-3

[25] Sheng W, Xi N, Song M, Chen Y (2001) Graph-based surface merging in CAD-guided dimensional inspection of automotive parts. In: IEEE int conf robot autom, vol 3. IEEE, pp 3127–3132. doi:10.1109/ROBOT.2001.933098

[26] Simon DA (1996) Fast and accurate shape-based registration. Ph.D. thesis, Carnegie Mellon University Pittsburgh

[27] Suwelack S, Röhl S, Bodenstedt S, Reichard D, Dillmann R, dos Santos T, Maier-Hein L, Wagner M, Wünscher J, Kenngott H, Müller BP, Speidel S (2014) Physics-based shape matching for intraoperative image guidance. Med Phys 41(11):111901. doi:10.1118/1.4896021 10.1118/1.4896021 25370634

[28] Thompson S, Totz J, Song Y, Johnsen S, Stoyanov D, Ourselin S, Gurusamy K, Schneider C, Davidson B, Hawkes D, Clarkson MJ (2015) Accuracy validation of an image guided laparoscopy system for liver resection. In: SPIE, vol 9415, pp 941, 509–941, 509–12. doi:10.1117/12.2080974

[29] Totz J, Thompson S, Stoyanov D, Gurusamy K, Davidson B, Hawkes DJ, Clarkson MJ (2014). Fast semi-dense surface reconstruction from stereoscopic video in laparoscopic surgery. IPCAI.

[30] Wang M, Song Z (2013). Optimal number and distribution of points selected on the vertebra for surface matching in CT-based spinal navigation. Comput Aided Surg.

[31] Wang M, Song Z (2016). How does adding anatomical landmarks as fiducial points in the point-matching registration of neuronavigation influence registration accuracy?. Comput Assist Surg.

